# Cholera toxin and O-specific polysaccharide immune responses after oral cholera vaccination with Dukoral in different age groups of Bangladeshi participants

**DOI:** 10.1128/msphere.00565-23

**Published:** 2024-02-23

**Authors:** Pinki Dash, Al Hakim, Aklima Akter, Hasan Al Banna, M. Hasanul Kaisar, Amena Aktar, Sultana Rownok Jahan, Jannatul Ferdous, Salima Raiyan Basher, Mohammad Kamruzzaman, Fahima Chowdhury, Afroza Akter, Imam Tauheed, Ana A. Weil, Richelle C. Charles, Stephen B. Calderwood, Edward T. Ryan, Regina C. LaRocque, Jason B. Harris, Taufiqur Rahman Bhuiyan, Firdausi Qadri

**Affiliations:** 1 Infectious Diseases Division, International Centre for Diarrhoeal Disease Research, Bangladesh (icddr,b), Dhaka, Bangladesh; 2 Department of Genetic Engineering and Biotechnology, Jagannath University, Dhaka, Bangladesh; 3 Department of Biochemistry and Molecular Biology, Mawlana Bhashani Science and Technology University, Santosh, Tangail, Bangladesh; 4 Department of Medicine, University of Washington, Seattle, Washington, USA; 5 Division of Infectious Diseases, Massachusetts General Hospital, Boston, Massachusetts, USA; 6 Department of Medicine, Harvard Medical School, Boston, Massachusetts, USA; 7 Department of Microbiology, Harvard Medical School, Boston, Massachusetts, USA; 8 Department of Immunology and Infectious Diseases, Harvard School of Public Health, Boston, Massachusetts, USA; 9 Division of Global Health, Massachusetts General Hospital for Children, Boston, Massachusetts, USA; 10 Department of Pediatrics, Harvard Medical School, Boston, Massachusetts, USA; University of Florida, Gainesville, Florida, USA

**Keywords:** O-specific polysaccharide, Dukoral, vibriocidal assay, antibody secreting cells

## Abstract

Vaccination is important to prevent cholera. There are limited data comparing anti-O-specific polysaccharide (OSP) and anti-cholera toxin-specific immune responses following oral whole-cell with cholera toxin B-subunit (WC-rBS) vaccine (Dukoral, Valneva) administration in different age groups. An understanding of the differences is relevant because young children are less well protected by oral cholera vaccines than older children and adults. We compared responses in 50 adults and 49 children (ages 2 to <18) who were administered two doses of WC-rBS at a standard 14-day interval. All age groups had significant IgA and IgG plasma-blast responses to the OSP and cholera toxin B-subunit (CtxB) antigens that peaked 7 days after vaccination. However, in adults and older children (ages 5 to <18), antibody responses directed at the OSP antigen were largely IgA and IgG, with a minimal IgM response, while younger children (ages 2 to <5) mounted significant increases in IgM with minimal increases in IgA and IgG antibody responses 30 days after vaccination. In adults, anti-OSP and CtxB memory B-cell responses were detected after completion of the vaccination series, while children only mounted CtxB-specific IgG memory B-cell responses and no OSP-memory B-cell responses. In summary, children and adults living in a cholera endemic area mounted different responses to the WC-rBS vaccine, which may be a result of more prior exposure to *Vibrio cholerae* in older participants. The absence of class-switched antibody responses and memory B-cell responses to OSP may explain why protection wanes more rapidly after vaccination in young children compared to older vaccinees.

**IMPORTANCE:**

Vaccination is an important strategy to prevent cholera. Though immune responses targeting the OSP of *V. cholerae* are believed to mediate protection against cholera, there are limited data on anti-OSP responses after vaccination in different age groups, which is important as young children are not well protected by current oral cholera vaccines. In this study, we found that adults mounted memory B-cell responses to OSP, which were not seen in children. Adults and older children mounted class-switched (IgG and IgA) serum antibody responses to OSP, which were not seen in young children who had only IgM responses to OSP. The lack of class-switched antibody responses and memory B-cell responses to OSP in younger participants may be due to lack of prior exposure to *V. cholerae* and could explain why protection wanes more rapidly after vaccination in young children.

## INTRODUCTION

Cholera, an acute life-threatening diarrheal disease, is caused by cholera toxin producing strains of the bacterium *Vibrio cholerae* (1). Ultimately, universal access to clean water and sanitation are the optimal solutions for the eradication of the disease (2). However, in the interim, the use of oral cholera vaccines (OCVs) is recommended in both epidemic and endemic settings, and is a pillar of the World Health Organization’s (WHO) (3, 4) strategy to decrease cholera deaths by 90% and to eradicate cholera in at least 20 countries by 2030 (5).

*V. cholerae* O-specific polysaccharide (OSP) is the primary determinant of lipopolysaccharide (LPS) antigen specificity (6), and OSP targeted immune responses play a dominant role in protective immunity against cholera (7–9). Among the three WHO pre-qualified and commercially available OCVs, whole-cell with cholera toxin B-subunit (WC-rBS) (Dukoral, Valneva) is a killed oral whole-cell cholera vaccine containing heat-inactivated *V. cholerae* O1 Inaba and Ogawa classical strains, and a formalin-inactivated *V. cholerae* O1 El Tor strain and Ogawa classical strain, along with the recombinant cholera toxin B-subunit (CtxB) (10). This vaccine was used in this study to assess immune responses since it includes both the immunodominant polysaccharide (OSP) and protein (CtxB) antigens of *V cholerae*.

Young children are particularly vulnerable to cholera but achieve lower protective efficacy and a shorter duration of protection against disease following oral cholera vaccination (11, 12). For example, in a meta-analysis of seven studies of killed OCV, the effectiveness of vaccination in young children was only 30%, less than half of the effectiveness in older children and adults (13). In contrast, there is no evidence that young children are less well protected following natural infection with *V. cholerae* (9, 14). It has been postulated that the reduced level of protection following OCV in young children may be due to an impaired primary response to OSP antigen in young children and, compared to older individuals, that children under 2 years of age have a lower ability to mount systemic responses to T cell-independent vaccine antigens (9, 15). This hypothesis is consistent with results from a previous study by Leung and colleagues, who found that in young children who received WC-rBS, the magnitude of the anti-OSP responses was decreased compared to children recovering from natural *V. cholerae* infection (9).

An alternative hypothesis is that diminished vaccine responses in young children may also reflect a lack of boosting of anamnestic responses resulting from prior exposure, which is more common with increasing age. In other words, in cholera endemic areas, adults and older children are more likely to have been previously exposed to *Vibrio cholerae* than younger children, and there are limited data on immune responses against OSP in young child following Dukoral vaccine administration.

To evaluate the differences in vaccine responses across age groups in more detail, we subdivided child groups into older and younger children, since young child are predominantly affected by cholera. In this study, we sought to gain a detailed understanding of the defect in the immune response of young children to OCV by evaluating a broader array of responses to both OSP and the dominant protein antigen CtxB in participants receiving Dukoral vaccine, which was previously not assessed. We compared responses across multiple B-cell compartments including an evaluation of circulating antibody-secreting cells (ASCs) and memory B-cell (MBC) responses to both antigens. ASCs (or plasma blasts) can be detected transiently in the circulation after infection or oral vaccination and provide an early window into the mucosal response (16). The magnitude of these responses on day 7 is predictive of antigen-specific duodenal plasma cells that last for approximately 6 months after cholera (17). MBCs develop following a variety of infections and immunizations and are the mediators of a rapid anamnestic immune response on re-exposure to the same antigen (18). Hence, to assess whether vaccination with WC-rBS broadly induced significant systemic and mucosal immunological response, we assessed vibriocidal antibody responses in plasma, antigen-specific plasma antibody responses, circulating ASC responses, and anti-*V*. *cholerae* MBC responses against *V. cholerae* O1 OSP and CtxB at different time intervals and compared responses among otherwise healthy Bangladeshi young children, older children, and adult recipients of WC-rBS.

## RESULTS

### Study participants

A total of 99 individuals were enrolled in this study. Among them, 50 were adults (≥18 years, median 33 years) and 49 were children (2 to <18 years, median 6 years). Children entered were subclassified into two different categories for some further analysis: older children (*n* = 25; 5 to <18 years, median 11 years) and younger children (*n* = 24; 2 to <5 years, median 3 years) (Table 1). Blood samples were available at all four time points for 97 participants (14 days before vaccination and 2, 7, and 44 days after the initial vaccination). For two participants, only blood samples on days −14, 2, and 7 were available.

**TABLE 1:** Demographic characteristics of the study participants

Characteristics		Total (*n* = 99)
Age (years), median (range)	Adult (*n* = 50)	33 (18–50)
	Older children (*n* = 25)	11 (5 to <18)
	Younger children (*n* = 24)	3 (2 to <5)
Sex, *n* (%)	Female	60 (61)
	Male	39 (39)
No. (%) of participants with blood type	O	36 (36.4)
	A	17 (17.2)
	B	34 (34.3)
	AB	12 (12.1)

### Plasma vibriocidal antibody responses

**Fig 1:**
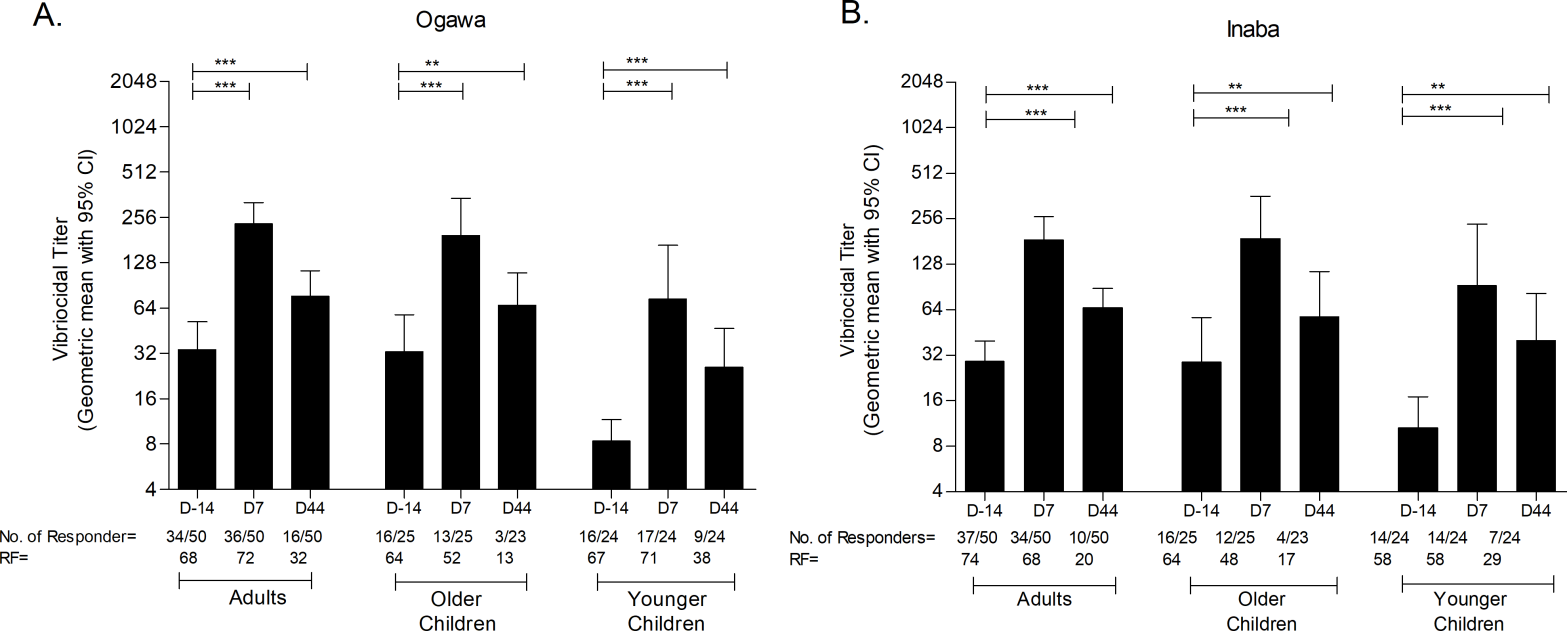
Plasma vibriocidal antibody responses in participants after oral cholera vaccination. Geometric mean titers (with 95% confidence interval) of vibriocidal responses to *V. cholerae* O1 Ogawa (**A**) and Inaba (**B**) in Bangladeshi participants receiving two doses of Dukoral vaccine separated by 14 days (days 0 and 14) are represented. Error bars indicate 95% confidence intervals. The Wilcoxon signed-rank test was used for analysis of the data. Asterisks denote statistically significant differences. **P < 0.01, ***P < 0.001. ns, not significant; RF, responder frequency.

Plasma vibriocidal antibody responses were measured in the vaccinees on days −14, 2, 7, and 44 in all three age groups. Vibriocidal antibody titers were significantly increased against both Ogawa and Inaba serotypes at each time point compared to both pre-vaccination day (day −14) and 2 days after the first dose of vaccination (day 2). Robust vibriocidal antibody responses were detected in all the age groups by day 7 (*P* < 0.001) after a single dose of WC-rBS vaccine, and these remained elevated at 1 month after the second dose of vaccine administration (day 44), though in older children the increase was not significant (Fig. 1A and B). Vibriocidal seroconversion was observed frequently from day 7 in all three age groups (Table 2).

**TABLE 2:** Vibriocidal seroconversion rate in WC-rBS vaccinees^*a*^

	D7/D14		D7/D2	
	Ogawa	Inaba	Ogawa	Inaba
Adult, percentage (number)	68 (34/50)	74 (37/50)	72 (36/50)	68 (34/50)
Older children, percentage (number)	64 (16/25)	64 (16/25)	52 (13/25)	48 (12/25)
Younger children, percentage (number)	67 (16/24)	58 (14/24)	71 (17/24)	58 (14/24)

^*a*^ D14, 14 days before vaccination; D2, 2 days after first vaccination; D7, 7 days after second vaccination.

### *V*. *cholerae* OSP Ogawa and CtxB-specific plasma antibody responses

**Fig 2:**
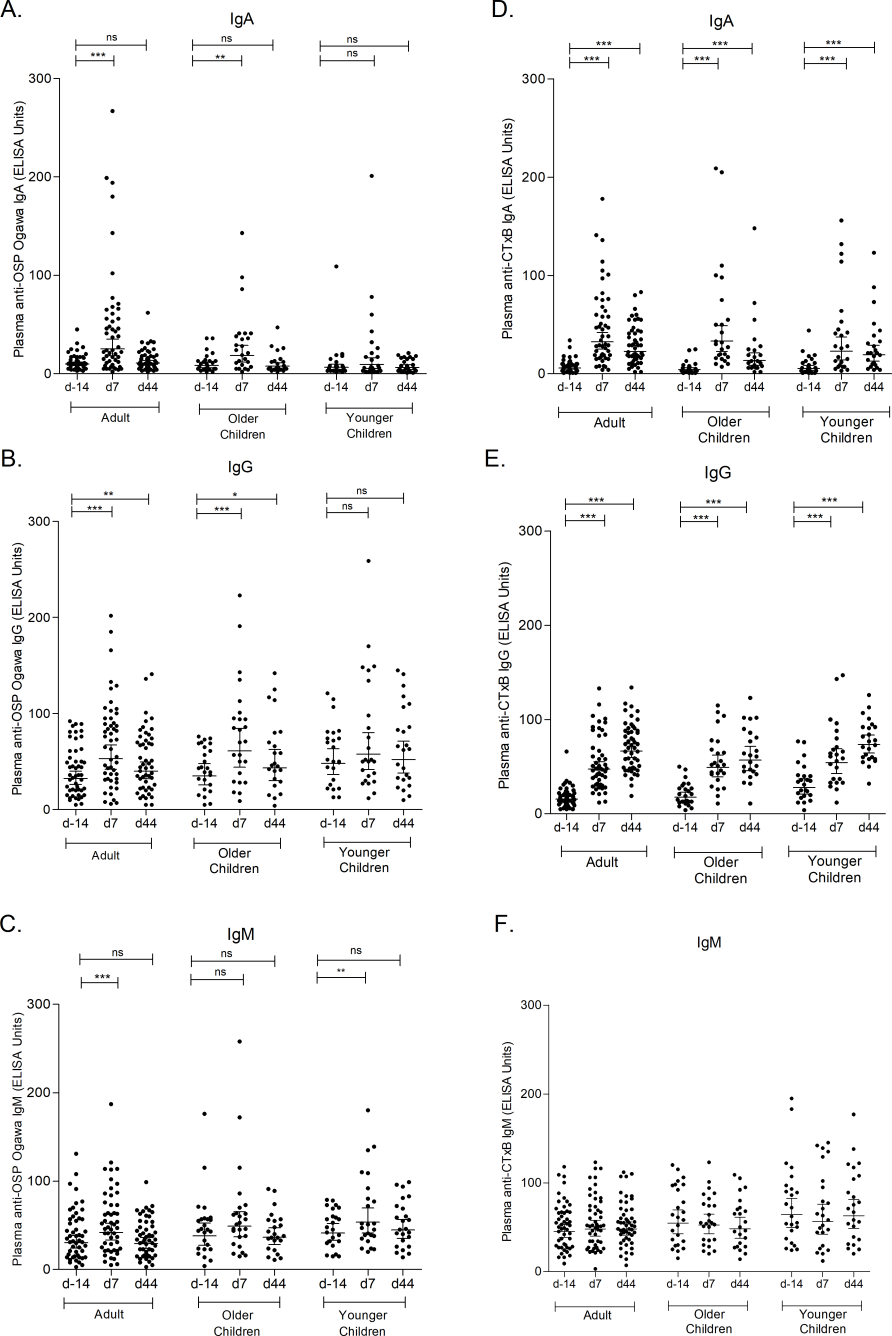
Plasma antibody responses in vaccinees to Ogawa OSP and CtxB (geometric mean with 95% confidence interval). Antibody responses to OSP Ogawa following vaccination in various age groups (**A**) Immunoglobulin A (IgA), (**B**) IgG, and (**C**) IgM and antibody responses to CtxB following vaccination in various age groups (**D**) IgA, (**E**) IgG, and (**F**) IgM. The Wilcoxon signed-rank test was used for analysis of the data. Each dot represents an individual plasma antibody titer; horizontal bars represent geometric means, and error bars indicate 95% confidence intervals. Asterisks indicate a statistically significant difference from baseline level. *P < 0.05, **P < 0.01, ***P < 0.001. CtxB, cholera toxin B-subunit; ELISA, enzyme-linked immunosorbent assay; ns, no significance; OSP, O-specific polysaccharide.

We analyzed plasma antibody responses to Ogawa OSP and CtxB in the vaccinees. In adult and older children, IgA and IgG responses specific for Ogawa OSP were significantly higher at day 7 after a single dose of the vaccine (*P* < 0.05) when compared to day −14. For younger children, a significant increase in an IgM response was detected at day 7 when compared to day −14 (Fig. 2A through C), but no significant IgA or IgG responses were seen. One month after the second dose of vaccination (day 44), IgG responses specific to OSP remained elevated in both adults and older children, whereas IgA responses specific to OSP had returned to baseline. In contrast, vaccinees in all three age categories had IgA and IgG plasma responses to CtxB that were significantly higher at days 7 and 44 than pre-vaccination (day −14) (Fig. 2D and E). As seen previously (15, 17), no significant IgM responses were observed against CtxB in any of the participants (Fig. 2F).

### Circulating ASC responses and flow cytometric analysis of plasma blasts after vaccination

**Fig 3:**
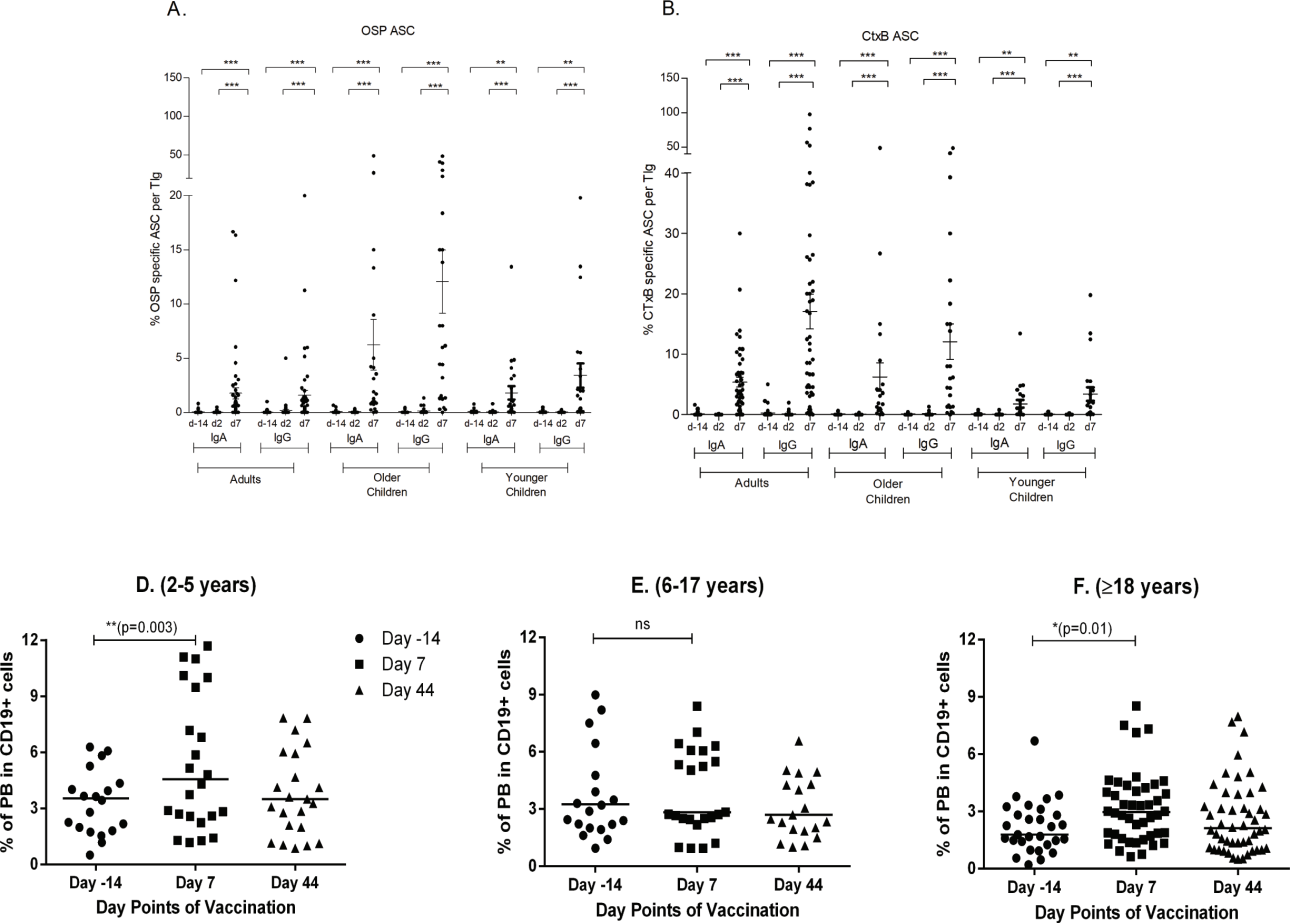
Ogawa OSP and CtxB-specific ASC responses and flow cytometric analysis of PBs in three age groups of vaccine recipients. Mean ± standard error of the mean of the fraction of circulating IgA and IgG ASC responses specific for OSP (**A**) and CtxB (**B**), out of total circulating IgA and IgG ASCs on the same days are represented. Circulating plasma blasts (**C–E**) as a proportion of circulating CD19^+^ cells on days −14, 7, and 44 after vaccination. Differences between groups were assessed using the paired parametric *t*-test. The Wilcoxon signed-rank test was used for analyses of the ASC data. Asterisks denote statistically significant differences. *P < 0.05, **P < 0.005, ***P < 0.001. ASC, antibody-secreting cell; CtxB, cholera toxin B-subunit; ns, no significance; OSP, O-specific polysaccharide; PB, plasma blast.

We detected OSP and CtxB-specific IgA and IgG ASC responses in peripheral blood mononuclear cells (PBMCs) isolated 7 days following vaccination in all three age groups, compared to days −14 and 2 (Fig. 3A and B). Similar patterns of IgA and IgG ASC responses in all the three groups of adults and older children (*P* < 0.001) and younger children (*P* < 0.005) specific to both Ogawa OSP and CtxB were observed on day 7 when compared to days −14 and 2. Corresponding with those data, the increase in circulating plasma blasts as a proportion of circulating CD19^+^ cells after vaccination was transient, peaking at day 7 and returning to baseline 30 days after completion of vaccination (Fig. 3C through E).

### OSP and CtxB-specific IgA and IgG MBC responses after vaccination

**Fig 4:**
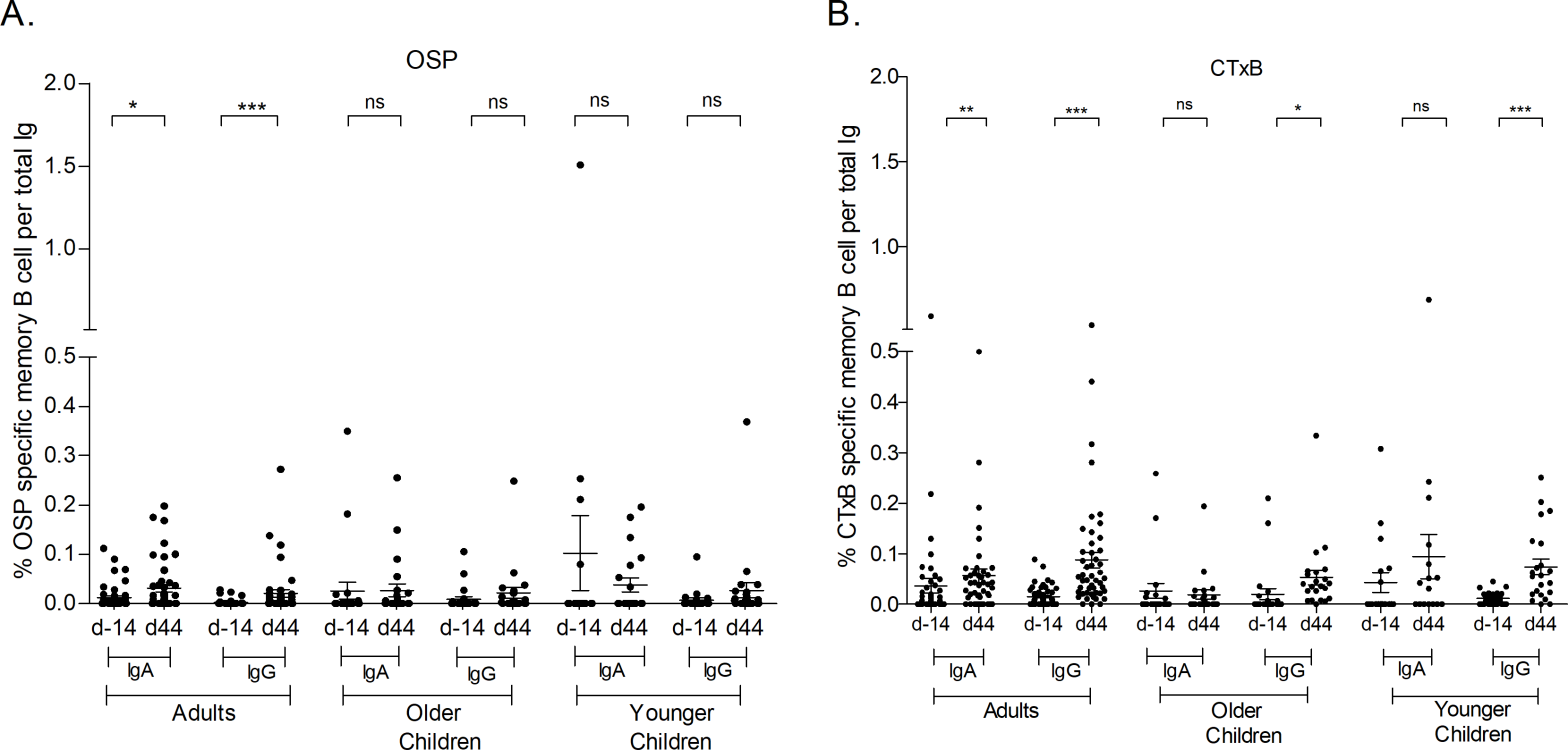
Ogawa OSP and CtxB- specific IgA and IgG memory B-cell responses in younger children, older children, and adult vaccinees. The figure shows mean antigen-specific IgA and IgG memory B cell responses to OSP ogawa (**A**) and CtxB (**B**), as a percentage of total memory B cells on indicated days, with error bars representing standard errors of the mean (mean ± standard error of the mean). The Wilcoxon signed-rank test was used for analysis of the data. Asterisks denote statistically significant differences. *P < 0.05, **P < 0.005, ***P < 0.001. ns, no significance.

We measured OSP and CtxB-specific MBC responses at days −14 and 44 in vaccine recipients (Fig. 4A and B). In adults, statistically significant increased anti-OSP Ogawa and anti-CtxB-specific IgA and IgG MBC responses were detected at day 44 compared to day −14. In both older and younger children, statistically significant increased IgG MBC responses to CtxB were detected at day 44 compared to day −14, whereas we did not find significant increases in anti-CtxB-specific IgA MBC or Ogawa OSP-specific IgA or IgG MBCs between these two time points.

### Comparison in vaccine responses between age groups

When vibriocidal titers were compared among the groups, baseline vibriocidal titer of younger children were significantly lower than that of adult and older children. Though vibriocidal titers of younger children at days 7 and 44 were higher than baseline, they remained significantly lower than those of adult and older children. Plasma anti-CtxB IgA responses were significantly higher at day 44 among adult and older children; then again, plasma anti-OSP IgA responses were higher at day 44 in adults than in younger children significantly; and at day 7, responses were significantly higher in older children than in younger children. Plasma anti-OSP IgM responses were significantly lower at day 44 in adults than in younger children. When ASC responses were compared among the groups, OSP-specific IgA and IgG responses were significantly higher in older children than in younger children at day 7, and CtxB-specific IgA and IgG responses were significantly higher in older children than in adults at day 7. We had only observed significant increased difference in CtxB IgG MBC response at day 44 among adult and older children (Fig. S1 to S4).

## DISCUSSION

This study provides a detailed, age-specific comparison of immune responses to two dominant antigens of *V. cholerae*, OSP and CtxB, in recipients of a killed oral cholera vaccine, WC-rBS. We observed several age-specific differences in the immune responses to this vaccine. Most importantly, older children and adults developed a class-switched circulating antibody response to OSP (IgA and IgG), while IgM responses to OSP and class-switched IgA or IgG did not reach statistical significance in young children. Furthermore, while adults had a significant IgA and IgG memory B-cell response to OSP, children failed to mount detectable MBC responses to OSP. This is the first study showing that while young children generate a significant increase in their vibriocidal antibody titer following the WC-rBS vaccine (similar to older vaccinees), they fail to generate class-switched antibody and memory B cell responses to OSP and CtxB following vaccination. This important evidence points to a possible reason why young children fail to develop equivalent protective immunity following inactivated oral cholera vaccines compared to older children and adults. These age-specific differences in the immune response to the WC-rBS vaccine are significant and are likely to explain the observation that although WC-rBS vaccine generates long-lasting protection in adults, protection wanes rapidly in young children, dropping from equivalent levels of protection to adults in the first year after vaccination to no protection 2 years after vaccination (19).

Interestingly, these results differ from the age-specific responses observed following natural infection with *V. cholerae*. A prior study of individuals recovering from cholera demonstrated that all age groups developed significant class-switched and MBC responses to *V. cholerae* O1 LPS (20). The fact that young children mounted class-switched and MBC responses to a canonically T cell-independent antigen in the setting of cholera but not following WC-rBS vaccination points to differences in the way the innate immune system is activated in response to cholera vaccination vs infection. Cholera holotoxin is a potent immunoadjuvant, while CtxB present in the OCV we evaluated lacks enzymatic activity. In other words, co-stimulation of the adaptive immune system provided by natural infection is sufficient to drive these memory responses to OSP, even in young children. Natural cholera infection is associated with induction of pro-inflammatory cytokines, while OCV results in a Treg response (21). This co-stimulation could be provided by mucosal-associated invariant T cells, which have previously been shown to be activated in cholera and likely aid in the production of LPS- and OSP-specific antibodies following cholera (22–24). Likewise, in a previous study by Saha and colleagues, post-vaccination vibriocidal titers were lower going from adults to older children to young children. Similar to our findings from this study, younger children receiving Shanchol vaccine also elicited similar immunological responses including vibriocidal titer and other LPS-specific plasma antibody responses (25). While findings were similar, the immunological breadth of this prior study was too limited to draw detailed conclusions about class-switched antibody and memory B-cell responses which were not assessed in the Shanchol.

It is unclear what mechanisms underlie the differences in age-specific responses to the WC-rBS vaccine. One possibility is that the development of class-switched and MBC responses with increasing age is dependent on repeated prior exposure to circulating *V. cholerae* in a population where the infectious disease is endemic. This hypothesis is supported by data from a non-endemic population that shows that repeated exposures are necessary to drive responses to *in vivo* expressed antigens and class-switched responses to the OSP antigen (26–28). Another, non-mutually exclusive possibility is that these age-specific differences are shaped by differences in the maturation of the adaptive immune system, especially given that older children and adults have a greater capacity to respond to T cell-independent antigens like OSP (9, 27).

No significant difference was observed in responses in the overall cohort by blood group, so this analysis has not been included for the sake of clarity and conciseness since the study is focused on age effects, and blood group would not appear to confound this analysis. The study was not adequately powered to look at sex differences within each age group; however, as expected and described previously (29), females had higher circulating levels of some antibodies. However, the distribution of male and female participants was similar across age groups, so we do not expect this to be a confounding factor in the analysis comparing responses between each age cohort, which is the major focus of the analysis.

Our study had some limitations. Most notably, since all the participants lived in an area where cholera is endemic, we were unable to control for previous and ongoing exposure to *V. cholerae*. However, this limitation is counterbalanced by the fact that vaccine studies conducted in a population where the disease is endemic also have greater clinical relevancy to disease prevention. Also, we compared our findings with prior studies of oral cholera vaccination in a non-endemic setting to better understand the role of repeated exposures in priming immune responses across ages. Second, we focused on the WC-rBS vaccine to compare the age-specific effects on a canonical T-cell independent (OSP) vs a T cell-dependent antigen (CtxB), yet these may differ from responses to the newer bivalent inactivated cholera vaccine, which is the main vaccine in the current global cholera vaccine stockpile. Hence, a similarly detailed comparison of long-term age-specific responses to OSP may be important in recipients of the bivalent vaccine as well.

In conclusion, we demonstrate that adults and older and younger children, vaccinated with two doses of the WC-rBS killed oral cholera vaccine, all mount a significant mucosal response to vaccination but that older children and adults have a more prominent class-switched antibody response, and older age is also associated with a robust OSP MBC response which is absent in children. These findings may explain age-specific differences in the duration of protection following oral cholera vaccination.

## MATERIALS AND METHODS

### Study design and sample collection from participants

**Fig 5:**
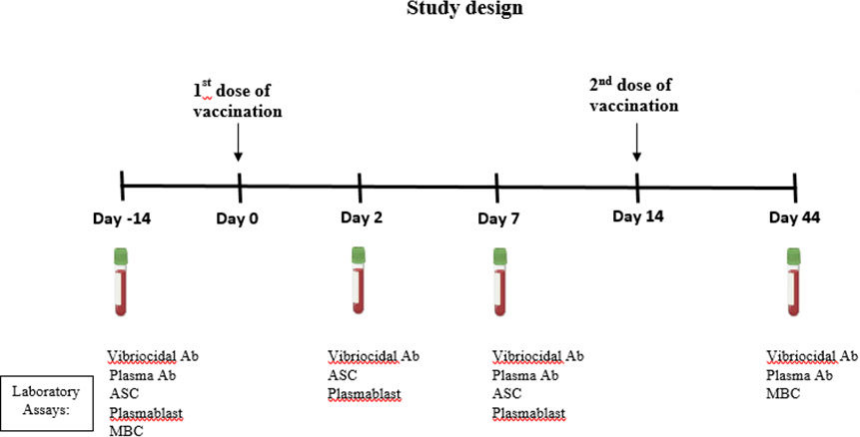
Study design: the figure outlines the design of the study. Participants were administered with two doses of the killed oral whole-cell cholera vaccine at a 2-week interval (at days 0 and 14). Blood was collected from the participants on days −14, 2, 7, and 44.

A total of 99 healthy volunteers (adults, *n* = 50; children, *n* = 49) were enrolled in this study. Participants were administered with two doses of the killed oral whole-cell cholera vaccine WC-rBS (Dukoral, Valneva) at a 2-week interval (at days 0 and 14). Blood was collected from the participants on days −14, 2, 7, and 44 (Fig. 5). At each time point, plasma samples were assayed for vibriocidal antibodies to *V. cholerae* O1 Ogawa and Inaba. At days −14, 7, and 44, plasma samples were also assayed for IgA, IgG, and IgM antibodies to Ogawa OSP and anti-CtxB. We also assessed circulating antigen-specific IgA and IgG ASCs at days −14, 2, and 7 in isolated PBMCs. Measurement of the proportion of circulating plasma blasts was performed using flow cytometry at days −14, 7, and 44. Antigen (OSP and CtxB)-specific IgA and IgG MBC levels were assessed at day −14 sample and day 44 sample.

### Isolation of PBMCs and plasma from whole blood

Sodium-heparinized blood was collected, and then by density gradient centrifugation using Ficoll-Isopaque (Pharmacia, Piscataway, NJ, USA), plasma and PBMCs were isolated (30, 31). To detect ASC responses through enzyme-linked immunospot (ELISPOT) assay, mononuclear cells (MNCs) were used and these MNCs were also cultured to detect MBC level as described below.

### Plasma vibriocidal antibody responses

Plasma vibriocidal antibody responses were assessed using two strains named *V. cholerae* O1 Ogawa (X-25049) and *V. cholerae* O1 Inaba (T-19479) as the target organisms at each time point as previously described (32, 33). Participants were considered as responders if they had a ≥4-fold increase (31) in vibriocidal titer observed at days 7 and 44 compared to their pre-vaccination day (day −14) titer.

### CtxB- and OSP-specific IgA, IgG, and IgM antibody responses

CtxB- and OSP-specific IgA, IgG, and IgM antibody responses were assessed in plasma at days −14, 7, and 44 as described previously using enzyme-linked immunosorbent assay (32, 34). Bovine serum albumin (BSA) (1 µg/mL), which was dissolved in bicarbonate buffer (pH 9.6–9.8) and with 0.3 nmol of ganglioside GM1/mL followed by recombinant CtxB subunit (0.5 µg/mL) (gift from Ann-Mari Svennerholm, University of Gothenburg, Sweden). Plasma (100 µL) was added per well. Anti-human IgG, IgA, or IgM (1:1,000 dilution; Jackson ImmunoResearch, West Grove, PA, USA), which were horseradish peroxidase-conjugated, was added to the wells as secondary antibodies, and plates were developed using ortho-phenylene diamine (Sigma-Aldrich, St. Louis, MO, USA) in 0.1 M sodium citrate buffer (pH 4.5) and 0.012% hydrogen peroxide as substrate. These plates were finally read kinetically at 450 nm for 5 minutes. Maximal rate of change in optical density was measured as milliabsorbance units per minute. Normalization was done by calculating the ratio of the test sample to a standard of pooled convalescent-phase plasma from previously infected cholera patients as a positive control (30, 31).

### ASC responses by ELISPOT

ASC responses were assessed through ELISPOT technique as described previously (35). Affinity-purified goat anti-human immunoglobulin (Jackson ImmunoResearch) as a positive control, *V. cholerae* O1 Ogawa OSP: BSA (10 µg/mL), GM1 ganglioside (3 nmol/mL) followed by recombinant CtxB (2.5 µg/mL), and as a negative control, keyhole limpet hemocyanin (KLH, 2.5 µg/mL; Pierce Biotechnology, Rockford, IL, USA) were used as coating antigen to coat nitrocellulose-bottom plates (Millipore, Bedford, MA, USA). Goat anti-human IgG (Southern Biotech, Birmingham, AL, USA) conjugated with alkaline phosphatase was used to detect IgG ASCs, and to detect IgA ASCs, horseradish peroxidase-conjugated goat anti-human IgA (Southern Biotech) was added to the plates, respectively. Plates were incubated overnight at 4°C, and on the following day, plates were developed using 5-bromo-4-chloro-3-indolylphosphate-nitroblue tetrazolium (Sigma-Aldrich) and 3-amino-9-ethylcarbazole (pre-mix solution, Sigma-Aldrich) as substrate, respectively.

### MBC responses

MBC assays were performed using PBMCs on days −14 and 44 as previously described (14, 36, 37). PBMCs (5 × 10^5^) were placed per well in cell culture plates (BD Biosciences, San Jose, CA, USA) which contained culture media including RPMI 1640, 10% fetal bovine serum (FBS), 2 mM L-glutamine, 200 units/mL penicillin, 200 mg/mL streptomycin, and 50 mM beta-mercaptoethanol. A mixture of three B-cell mitogens containing 6-µg/mL CpG oligonucleotide (Operon, Huntsville, AL, USA), a 1/100,000 dilution of crude pokeweed mitogen extract, and a 1/10,000 dilution of fixed *Staphylococcus aureus* Cowan (Sigma-Aldrich) was added for stimulating antigen-independent proliferation and differentiation of MBCs into ASCs. For the MBC assay, plates were coated with antigens named affinity-purified goat anti-human immunoglobulin, OSP, CtxB, or KLH as described for the ASC assay earlier. Respectively, 20% and 80% of the cells from each well of the culture plate were added for detection of total ASC and antigen-specific ASC of that isotype. Proper stimulation of PBMC was defined as a threefold increase in the number of total MBCs after stimulation compared to un-stimulated cells. Exclusion criteria of our analysis were any of the following reasons as previously described: total MBC specimen did not have enough stimulation; the study participant’s sample had four or more antigen-specific ASC spots in the same sample prior to stimulation; participant samples had four or more ASC spots to the negative control antigen (14, 20, 31).

### Analytical flow cytometry

All immunophenotyping experiments were performed on ARIA III instruments (Becton Dickinson). Separated PBMCs were incubated with the following fluorochrome-tagged antibodies: anti-CD3 Pacific Blue, anti-CD38 PE, anti-CD20 PECy7 (BD Biosciences), anti-CD19 FITC (BD Pharmingen) and anti-CD27 APC (eBioscience) as described previously (16). The cells were stained, incubated, washed, and acquired with a FACSAria III cytometer (BD Biosciences) using the FACSDiva software program. The plasma blasts were gated as CD3^−^ CD19^+^ CD20^−/low^ CD27^high^ and CD38^high^. The cells were gated on an extended lymphocyte gate to include blasting cells. We analyzed the acquired data with FlowJo software (version 10.6.1; TreeStar Inc., USA), separating the lymphocyte population based on side and forward scattered light. The results are expressed as percentages.

### Statistical analysis

Data analysis and figures were generated using Graphpad Prism (version 6.0; GraphPad Software, Inc., La Jolla, CA, USA). We used Wilcoxon signed-rank *t*-tests and Mann-Whitney *U* tests to assess the differences in the magnitude of responses within a group and among the groups, respectively. All reported *P* values were two tailed, with a cutoff of *P* ≤ 0.05 considered a threshold for statistical significance.

## Data Availability

The raw data supporting the findings of this article will be made fully available by the authors, without undue reservation.

## SUPPLEMENTAL MATERIAL

Fig. S1 msphere.00565-23-s0001.tif Plasma vibriocidal antibody responses after oral cholera vaccination among the three groups of participants.

Fig. S2 msphere.00565-23-s0002.tif Plasma antibody responses among the three age groups of vaccinees to Ogawa O-specific polysaccharide (OSP) and cholera toxin (CtxB).

Fig. S3 msphere.00565-23-s0003.tif Ogawa O-specific polysaccharide (OSP) and cholera toxin (CtxB) specific antibody secreting cell (ASC) responses among the three age groups of vaccine recipients.

Fig. S4 msphere.00565-23-s0004.tif Ogawa OSP and CtxB- specific IgA and IgG memory B cell responses among younger children, older children, and adult vaccinees.

ASM does not own the copyrights to Supplemental Material that may be linked to, or accessed through, an article. The authors have granted ASM a non-exclusive, world-wide license to publish the Supplemental Material files. Please contact the corresponding author directly for reuse.
