# Supplementary figures and images for: Cholera toxin and O-specific polysaccharide immune responses after oral cholera vaccination with Dukoral in different age groups of Bangladeshi participants
Source: mSphere. 2024 Feb 23;9(3):e00565-23. doi: 10.1128/msphere.00565-23 (PMC10964428; doi:10.1128/msphere.00565-23)

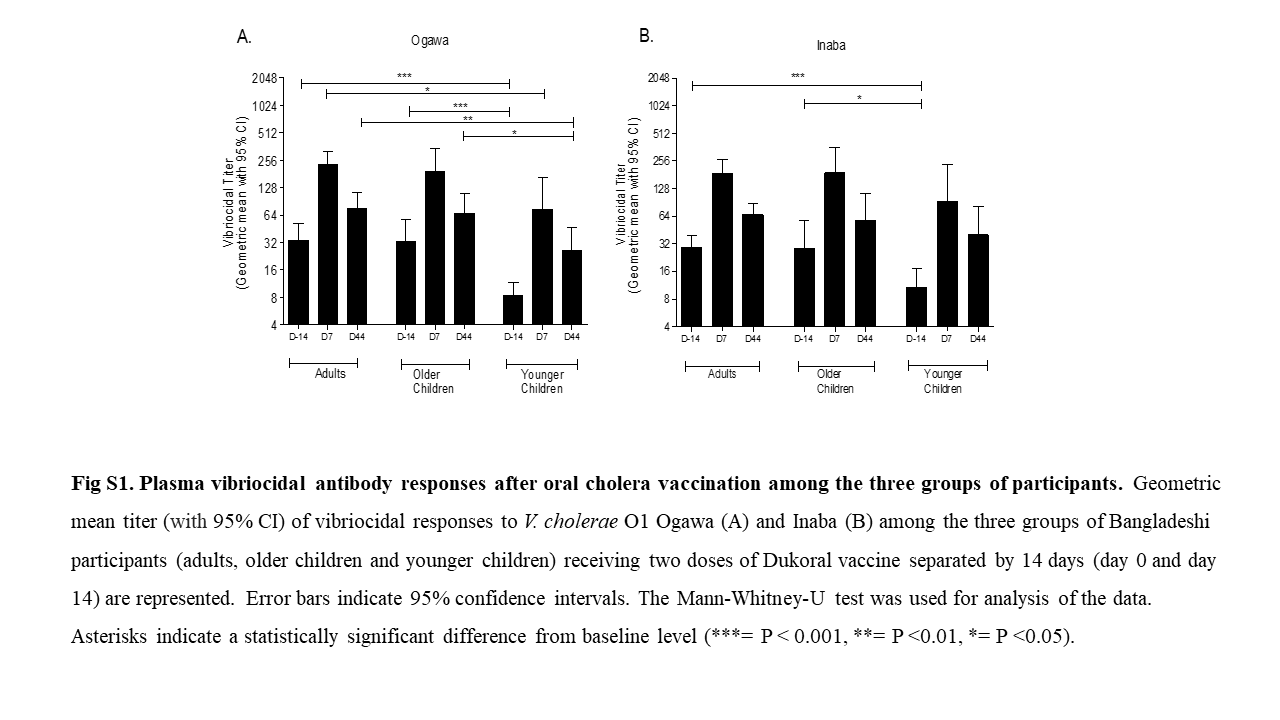

Supplement: Fig. S1 — Plasma vibriocidal antibody responses after oral cholera vaccination among the three groups of participants. [file msphere.00565-23-s0001.tif]

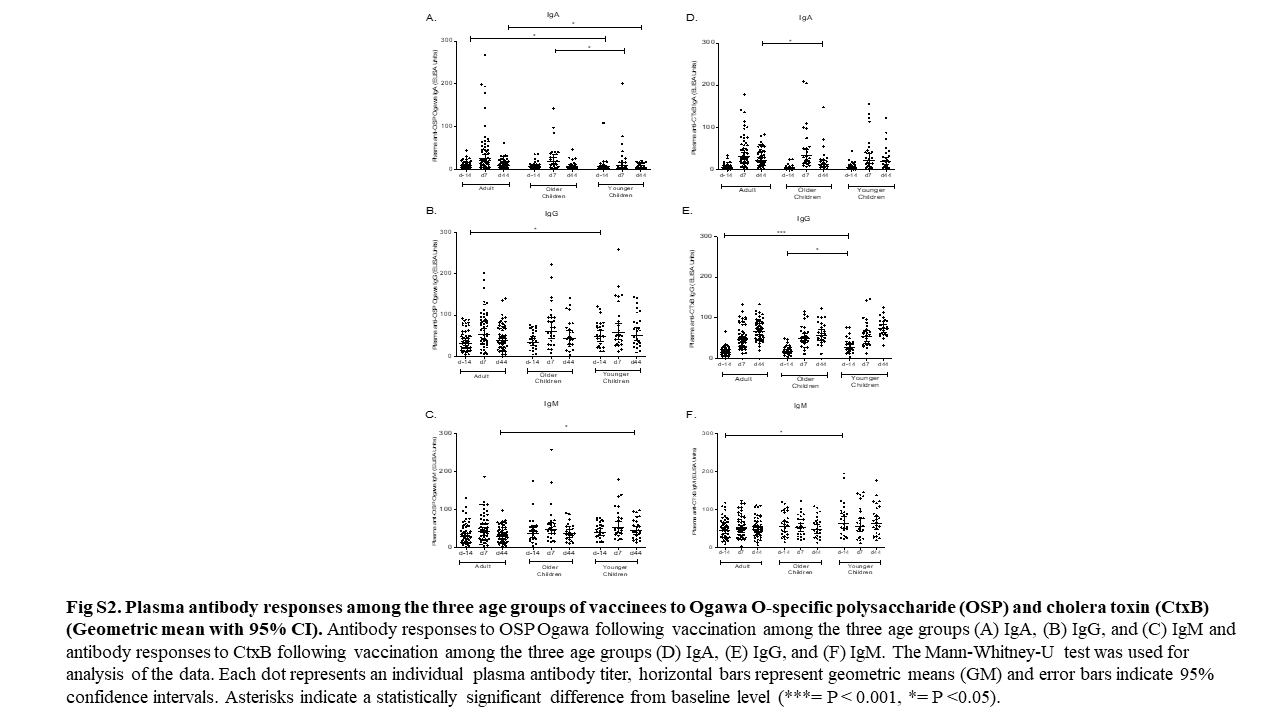

Supplement: Fig. S2 — Plasma antibody responses among the three age groups of vaccinees to Ogawa O-specific polysaccharide (OSP) and cholera toxin (CtxB). [file msphere.00565-23-s0002.tif]

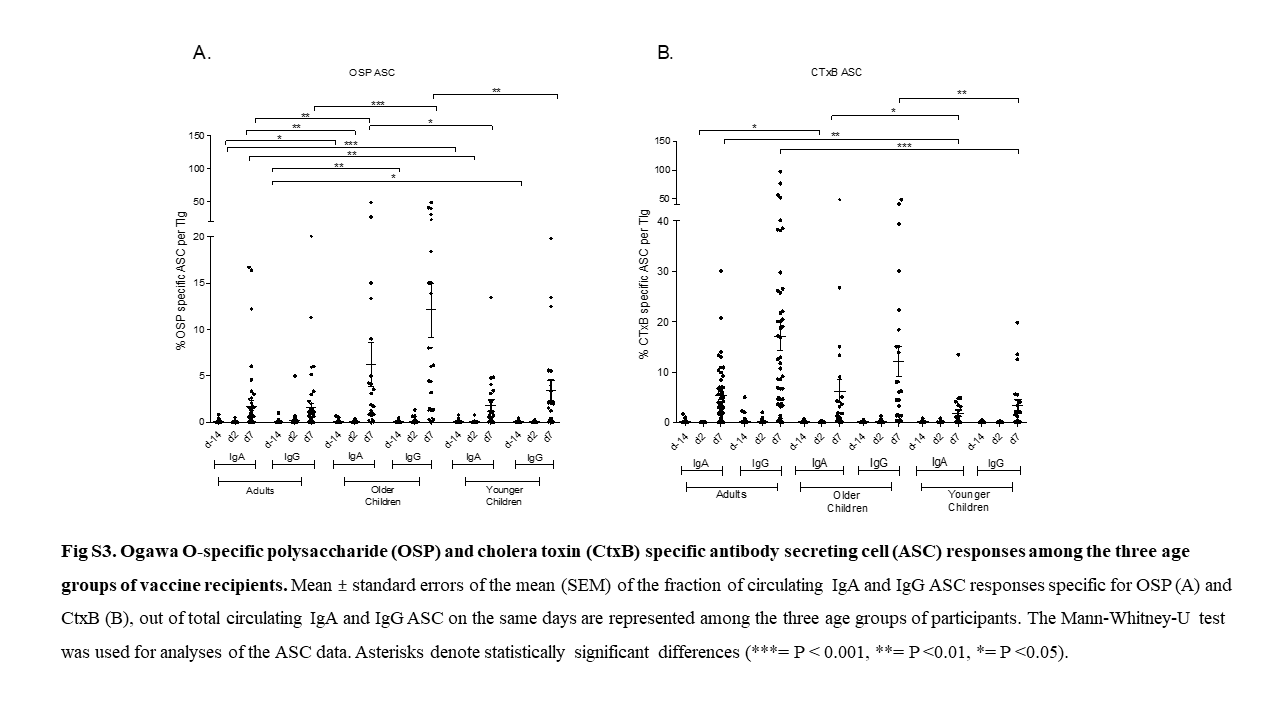

Supplement: Fig. S3 — Ogawa O-specific polysaccharide (OSP) and cholera toxin (CtxB) specific antibody secreting cell (ASC) responses among the three age groups of vaccine recipients. [file msphere.00565-23-s0003.tif]

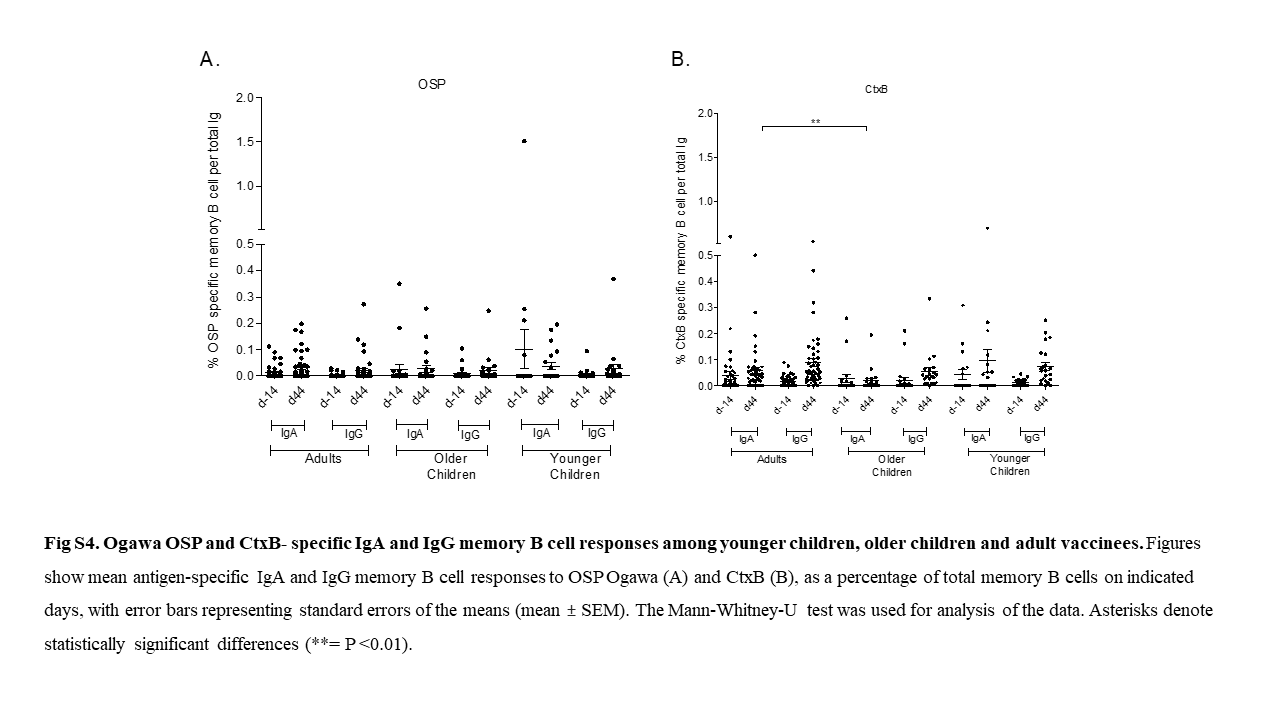

Supplement: Fig. S4 — Ogawa OSP and CtxB- specific IgA and IgG memory B cell responses among younger children, older children, and adult vaccinees. [file msphere.00565-23-s0004.tif]
